# Monthly Summary

**Published:** 1877-08

**Authors:** 


### MONTHLY SUMMARY.

*Excision of the Inferior Maxilla.*—Dr. J. W. S. Gouley, of New York, reporter, presented a report of a case of subperiosteal excision of the entire inferior maxillary bone, and solicited from American and foreign surgeons records of their unpublished cases, to add them to those he has already collected for analysis in an essay he is now preparing. The doctor's patient was a female nineteen years old, with phosphorus necrosis of four years' standing, involving the entire lower jaw, made up of an enormous involucrum, rendering mastication impossible. March 19, 1864, the patient having been etherized, the entire jaw was removed by external incision, and she made an excellent recovery. When last seen, seven and a half years after the operation, the bone reproduction extended from the body to the rami of the jaw.

He said it was not common in phosphor-necrosis to find an involucrum, but in his case there was one, while there was none of the pumice-stone deposit of Stanly. He explained this by the violent phlegmonous periostitis which occurred a year previous.

As regards various operations, chiefly modifications of the external incision, he wished particularly to call attention to the intrabuccal, which offers many advantages.

The operation for removal of the lower maxilla was usually accomplished at two or more sittings, but in his list he included as one all which were merely continuations of the same.

Dr. Hutchinson, of Brooklyn, said in malignant tumors he should consider the intrabuccal operation as an unsafe one—there would not be the same certainty of complete removal; but for phosphor-necrosis he considered it perfectly feasible and easy. It was also applicable to removals of the superior maxilla.

Dr. Hyde said the incision must depend largely on the requirements of the case. He had usually removed sequestrum from the inside.

Dr. Gouley, in reply to a question by Mr. Lister, stated that the bone was entirely dead, and should have been removed two years earlier.

Prof. Lister thought the incision should be determined by the tumor. He certainly would not employ the intrabuccal one in malignant cases, but might in innocent ones; although even here in males the scar could be well concealed, and even in females was not serious if made behind the ramus.—*Med. Record Inter. Med. Congress.*

---

*Excision of the Branches of the Fifth Pair in Neuralgia.*—Prof. Weinlechner, in a communication to the Vienna Medical Society (*Wien. Med. Woch.*, Jan. 20,) stated that he had performed excision of the branches of the trigeminus in twenty cases of severe neuralgia. He had found the operation much more successful when the neuralgia was limited in extent than when it was diffused. The deeper and more central was the resection, the more certain was the relief of the neuralgia; but in very deep excisions we are deprived of the possibility of again resorting to the operation in case a relapse might render this desirable. Comparing this operation with that of tying the common carotid, we may say that in very obstinate and painful cases both modes of procedure may produce a temporary or permanent cure; but neither method is without danger, as they both are sometimes followed by pyæmia, erysipelas, etc. In general the ligature of the artery is the easier operation of the two; but in old persons there is the danger of atheromatous degeneration, while in the younger ones, if the ligature is followed by hemorrhage, severe disturbances of the cerebral nutrition may ensue. As a general rule it may be stated that neurectomy is to be preferred in limited neuralgia, while in diffused neuralgia with vascular dilatation, ligature of the carotid should be performed.—*Med. Times and Gaz.*

---

*The Length of the Frœnum of the Tongue*—M. Baily (*Journal des Sages-femmes*) remarks that, instead of the condition known as tongue-tie preventing the child from sucking, it aids it in the performance of that function. It is but a vulgar error to suppose that tongue-tie is the cause of the child being unable to seize the teat. On the contrary, M. Bailly has seen a case where the tongue was perfectly free, unbridled by a frænum. The child in this case was unable to take the breast, but had to be fed by the bottle, because its long unbridled tongue was glued to the roof of its mouth. The child was unable to depress the tongue, and seize the nipple.—*London Med. Record.*

---

*The Treatment of Atheromatous Cysts of the Neck.*—Esmarch recommends in those forms of atheromatous cysts of the neck which can only be removed with difficulty, or with the formation of a large cicatrix, puncture of the sac, the injection of a one per cent. solution of carbolic acid, until the solution returns clear, and then the injection of a solution of a Lugol's solution, containing about three per cent. of iodine, and iodide of potassium in water, which he allows to flow out again after the lapse of a few minutes. If the tumor have not considerably diminished in size in the course of six or eight weeks the operation is repeated. In the course of half a year the cyst is usually reduced to the size of a small node.—*Med. and Surg. Reporter.*

*Injections of Carbolic Acid for Neuralgia.*—DR. MERTEN, of Neuwedell, reports (*Allgem. Med. Cent. Zeit.*, Sept. 6. 1877) four cases of severe Neuralgia that were cured with surprising rapidity by one or two hypodermic injections of carbolic acid. In the first case, the patient, a hysterical young woman, had suffered for several months from strabismus and ptosis of the field of vision in the same eye by almost one-half. Ophthalmoscopic examination gave negative results. She was suddenly seized with intense neuralgia of the left malar nerve, for which twenty drops of a 2 per cent. solution of carbolic acid was injected over the cheek-bone. It caused at first intense burning pain, with redness and swelling at the point of injection, but one hour later all pain had disappeared. On the next morning there was no pain at all, the ptosis and strabismus had entirely disappeared, and the field of vision of the left eye had markedly increased. Unfortunately, the patient did not again present herself for observation. The second case was one of acute oöphoritis with severe neuralgia of the external cutaneous nerve of the thigh, and intense pain about the anus on defecation. Two injections of a syringeful of the 2 per cent. acid solution permanently relieved all the pains. The third case was one of suppressio memsium and sciatica. Two hours after injection of a syringeful of the above solution the pain ceased entirely and did not return. Menstruation was subsequently regular. The last case was one of myalgia of the right side of the neck, consequent on exposure to cold. A single injection of the 2 per cent. solution produced a cure.—*Practitioner.*

---

*Anœsthesia.*—Dr. Addinell Hewson, of Philadelphia, read a paper on "Anæsthesia, as produced by Nitrous Oxide and rapid breathing."

He stated the method had been suggested to him by W. G. Bonwill, a dentist of Philadelphia. and he had tried it in a number of cases, in public and private practice, with varying but satisfactory results. The method pursued was to cause the patient to breathe from forty to fifty times in the minute, the effect of which in from three to five minutes was a tingling of the surface, with flush face. Consciousness remained unimpaired and the patient would perform any act desired. but was rendered totally devoid of sensibility. This method would be advantageous for short minor operations, especially those about the nose, throat, etc. The process occupied a longer time in young people and in cold weather. He explained the theory of its action by the retention in the blood of carbonic acid.—*Med. Record. Inter. Med. Congress.*

*On Local Anæsthesia by Bromhydrate of Quinine.*—Dr. Thaon (*Nice Medicale,* January, 1877) has observed a case of intermittent fever which resisted the administratisn of bromhydrate of quinine administered internally, or by the hypodermic method. Eighty centigramms, and even one gramme, were given in this way for several days without destroying the fever; but one of the punctures made on the forearm towards the external border at four finger-breadths below the elbow and probably at the level of one of the anterior branches of the musculo-cutaneons nerve, produced complete anæsthesia of the skin over a spot nearly five inches long by two and a-half inches in breadth. The loss of perception of heat was somewhat more extensive. One month after the puncture, this anæthesia remained almost as considerable as at the commencement. Dr. Thaon thinks that some application may be made of this fact to the treatment of neuralgia.—*London Med. Record.*

---

*Bromide of Potassium as a Caustic.*—In a paper read at the recent meeting of the French Association for the Advancement of Science, M. Peyrand, of Dibourne, claims for bromide of potassium certain properties hitherto but slightly recognized—properties which will extend already wide range of the therapeutical uses of this salt. He found that subcutaneous injection in rabbits of concentrated solutions of the salt led to sloughing of the skin, and from this he was led to try the value of what he considered to be the escharotic properties of bromide of potassium upon malignant and other growths, either by means of injections into the tumour or by the application of the powdered salt to a raw surface. The action of the salt is completely resisted by the tegument. His first clinical experiment on the subject took place in April, 1874, when, by means of daily applications of powdered bromide, he affected the removal within twenty-eight days of an epitheliomatous growth on the face. He has since had equally good results from this treatment of atonic ulcers of the leg, rapid cicatrization following the separation of sloughs produced by the application. In such cases he uses either the powder or an ointment of one part in five, or a mixture (one in ten) of glycerine and the bromide. In many skin affections, as chronic eczema, pityriasis, and acne, in phagedæna, ulcerative stomatitis, and many other local inflammatory disorders, he has found it of use. As a local hæmostatic, a solution of one in fifty has served for epistaxis, and as a general hæmostatic its success in many cases of hæmoptysis and metorrhagia was very marked, where ergot, perchloride of iron, and rhatany had failed.—*Lancet.*

*Death during Etherization.*—Dr. Benjamin W. Robinson, of Fayetteville, N, C., reports (*Virginta Med. Monthly*, April, 1877) the case of Mrs. McNeil, who consulted him on account of a tumour of the breast, which he extirpated while the patient was under the influence of ether. The tumour returned, and the operation repeated in ten months. Again after the lapse of six months, a third operation was performed, and Squibs ether was administered in a conical inhaler. During the inhalation the pulse improved in volume and force. In about twenty minutes after the operation was begun. it was announced that, with gradually increasing pallous, the radial and temporal pulse which had been failing since the operation began, were extinct, and that the respiration was irregular. Brandy was administered subcutaneously, the foot of the operating table raised, and artificial respiration practised. The lapse of a few minutes promised thorough resuscitation, the patient became conscious, the horizontal posture was restored, and the operation was continued without the inhalation of any more ether. In a few minutes the patient vomited, after which it was found that she was sinking. All efforts at resuscitation now proved unavailing. A post-mortem examination appears not to have been made, but the history of the case leads to the suspicion that death was caused by the trachea becoming choked up with vomiting matter. —*Med. News and Library.*

---

*Phenicated Camphor.*—Under this title Dr. Saulez describes (*Bull. de Therap.*, August 30) a combination of carbolic acid and camphor, which exerts a remarkable influence on wounds of an unhealthy character, as proved by him in the Romarantin Hospital. The acid will take up a large quantity of camphor, but Dr. Saulez ordinarily employs a solution of two grammes and a half of the powder in one of the acid (nine grammes to one of alcohol,) which produces a pale yellow oleaginous liquid, in which the disagreeable odour of the acid is no longer to be recognized. It is not miscible with water or glycerine, and in employing it we have first to emulsify it by the addition of a twentieth part of the phenicated camphor either to olive oil or infusion of saponaria.—*Med. Times and Gaz.*

---

*Poisonous Properties of Glycerine,*—From a series of experiments, made by the subcutaneous injection of glycerine in dogs, Drs. DujardinsBeaumetz and Audige arrived at the following conclusions: I, Chemically pure glycerine may induce in the dog, within twenty-four hours, when introduced under the skin fatal accidents if employed in the dose of eight grammes to 1000 grammes of the weight of the animal. 2. The general characters of such poisoning accidents (acute glycerism) resemble within certain limits, those of acute alcoholism. 3. The necroscopical lesions in glycerinism are analogous to those of alcoholism, whence it would appear that the toxical action of the two bodies is very much the same. 4. In a therapeutical point of view, the introduction of too large a quantity of glycerine into the system is, probably, not without some danger.—*Med. Times nnd Gaz.* from *Bull. de Therap.*

---

*Death from Chloroform.*—Three deaths from the inhalation of chloroform are reported in the London weeklies for September 23. In two of them the history was that so common in such cases—violent struggling, stoppage of the pulse, death, and fatty degeneration of the heart discovered afterwards. The third case was the result of the inhalation when the stomach was full of food. Vomiting was followed by a deep inspiration, and the trachea and larynx filled with half-digested food, so that even tracheotomy did not restore the power of breathing. —*Med. News and Library.*

---

*Suppression of the Salivary Secretion.*—(*Can. Med. and Surg. Jour.*, April, 1877.] Dr. D. Baynes relates the details of one of those rare cases occurring after an attack of acute tonsillitis. Both Steno's and Wharton's ducts were found open on examination. The patient said that his tongue felt too large for his mouth, and that the latter seemed filled with tallow. He was continuously obliged to wash his mouth or drink both night and day to prevent the choking sensation experienced from the dryness of the mucous membranes. After three weeks' ineffectual treatment by stimulating gargles and internal remedies, a copious flow of saliva was induced by passing for ten minutes the frequently reversed current of a 12 cell zinccarbon galvanic battery through the parotid gland, the negative pole being connected to a probe placed in Steno's duct, while the positive was applied to the nape of the neck.—*Detroit Med. Journal.*

---

*Nussbaum's Narcosis.*—[*Neues Rep. of Pharm.*, 1876, *New Remedies*, Mar. 15, 1877.] The peculiar state called Nuss baum's narcosis, produced by the subcutaneous administration of a few centigrammes of morphia, about fifteen minutes previous to placing a patient under the influence of chloroform has already been known for some time, and made use of with great benefit during operations in the mouth or in fauces, as the full anæsthetic effects of the chloroform are preserved while the loss of consciousness is by no means complete. Still better results have lately been obtained by substituting a subcutaneous injection of a few centigrammes of muriate of narceine for the morphia. The hypodermic solution is best made as follows: 0.3 gm. of muriate of narceine are mixed with 20 gm. of distilled water in a flint or test-tube; the latter is placed in a water motor and heated until the salt is dissolved.—*New Remedies* 1877.

---

*The Russian Female Medical School.*—This, at the present time, comprises for the five years 430 students, of whom seventy-three are Israelites, nineteen Polish Catholics, eleven Polish Protestants, and the rest Orthodox. In order to belong to the school, women must attend the regular course of lectures, and undergo special examinations. All the Governments of the empire furnish students for this school. They belong to the middle classes, and are generally from twenty to twenty-five years of age, very few having passed their thirtieth year. Among them are sixty-eight married women.—*Med. and Surg. Reporter.*

---

*Properties of the Human Gastric Juice.*—M. Charles Richet has been studying these matters upon the person of the patient on whom Verneuil successfully performed gastrotomy. He has reached the following conclusions: 1. The acidity of the gastric juice, whether pure or mixed with food, is equivalent to 1.7 grammes of hydrochloric acid to a thousand grammes of fluid. 2. Acidity increases slightly at the end of digestion, and is independent of the quantity of liquid contained in the stomach. Wine and alcohol increase, but cane-sugar diminishes it. 3. If acid or alkaline matters are introduced, the gastric juice tends to return to its normal acidity. 4. The mean duration of digestion is from three to four and a half hours or more. Food does not pass successively, but in masses 5. According to four analyses made by a modification of Schmidt's method, it was proved that free hydrochloric acid exists in the gastric juice. 6. It is possible to extract all the lactic acid contained in the stomach, and to prove that there is one part lactic acid to nine parts hydrochloric acid. 7. Following the method of Berthelot, that is, by agitation with anhydrous ether and deprived of alcohol, it can be shown that lactic acid is free in the gastric juice. 8. The question so long in controversy as to the nature of the free acid in the stomach seems almost solved, and it may be said that in every 1,000 grammes of gastric juice there are 1.53 grammes of hydrochloric acid and 0.43 of lactric acid.—*Lyon Medical,* May 13, 1877,

